# Changes in ruminal and reticular pH and bacterial communities in Holstein cattle fed a high-grain diet

**DOI:** 10.1186/s12917-018-1637-3

**Published:** 2018-10-12

**Authors:** Yo-Han Kim, Rie Nagata, Akira Ohkubo, Natsuki Ohtani, Shiro Kushibiki, Toshihiro Ichijo, Shigeru Sato

**Affiliations:** 1 United Graduate School of Veterinary Science, Gifu University, Gifu, 501-1193 Japan; 2 Cooperative Department of Veterinary Medicine, Faculty of Agriculture, Iwate University, Morioka, Iwate 020-8550 Japan; 3 National Institute of Livestock and Grassland Science, Tsukuba, Ibaraki 305-0901 Japan

**Keywords:** Microbiota, pH, Reticulum, Rumen, SARA

## Abstract

**Background:**

Subacute ruminal acidosis (SARA) is characterized by a ruminal pH depression, and microbiota can also be affected by a higher acidity and/or dietary changes. Previous studies have revealed similar patterns in pH reduction in the rumen and reticulum, whereas changes in reticular pH and bacterial community following a high-grain diet are not fully understood. The aim of this study was to investigate the changes in reticular pH and bacterial community structure following a high-grain diet simultaneously with those in the rumen.

**Results:**

SARA was diagnosed when ruminal and reticular pH remained under 5.6 for 350 ± 14 and 312 ± 24 min/d, respectively, on the last day of the CON period. During the CON period, lower proportion of acetic acid and higher proportion of butyric acid were observed compared with the HAY period. The proportions of acetic acid and propionic acid were lower and higher, respectively, in the rumen compared with the reticulum. From 454 pyrosequencing analysis, the relative abundance of several genera differed significantly between the two periods and the two locations. During the HAY period, higher relative abundances of *Prevotella*, *Eubacterium*, *Oscillibacter*, and *Succiniclasticum* and lower relative abundances of *Ruminococcus*, *Clostridium*, and *Olsenella* were identified compared with the CON period. Furthermore, the relative abundance of *Eubacterium* was lower in the rumen compared with the reticulum. Bacterial diversity indices were significantly different between the HAY and CON periods, being higher in the HAY period. The quantitative real-time PCR showed that the copy numbers of several cellulolytic bacteria (*Fibrobacter succinogenes* and *Ruminococcus albus*) were higher during the HAY period.

**Conclusion:**

A high-grain diet showed similar impacts on the pH, fermentation, and bacterial community structure in the rumen and reticulum. During the CON period, ruminal and reticular pH decreased following the high-grain challenge, and lower bacterial diversity and changes in the bacterial composition, similarity, and bacterial copy numbers were observed due to a higher acidity and dietary changes compared with the HAY period. These changes may influence the fermentative ability of the rumen and reticulum.

**Electronic supplementary material:**

The online version of this article (10.1186/s12917-018-1637-3) contains supplementary material, which is available to authorized users.

## Background

Subacute ruminal acidosis (SARA) is a common health and production problem in dairy herds, and is defined as a condition characterized by ruminal pH < 5.6 for more than 3 h per day [1]. Monitoring of forestomach pH has been suggested as a potentially valuable tool for diagnosing SARA [2, 3]. Sato et al. [2] measured ruminal and reticular pH simultaneously using a radio transmission pH measurement system and observed similar patterns in pH reduction in the rumen and reticulum after feeding. More recently, Falk et al. [3] compared the ruminal and reticular pH of cows in early stages of lactation. They found that reticular pH was higher than ruminal pH in averaged pH profiles at all weeks of lactation. Collectively, these two studies demonstrated that reticular pH is closely correlated with ruminal pH.

Pyrosequencing technology is an alternative to traditional gel-based sequencing techniques for de novo DNA sequencing, and has been used to analyze ruminant gastrointestinal microbiota, especially in the rumen [4–6]. A decrease in ruminal pH following a high-grain diet is quite common; microbiota and epithelial bacterial communities in the rumen can also change due to higher acidity and/or dietary changes [5, 7]. Sato [5] reported that a high-grain diet caused not only decreased ruminal pH but also a reduction in the relative abundance of the ruminal phyla *Bacteroidetes* and *Proteobacteria* in cattle with repeatedly induced SARA. Furthermore, the addition of starch source reduces bacterial diversity indices [8, 9], and the relative abundances of ruminal phyla are affected by starch and oil additions [4].

Although rumen microbiota are influenced by rumen conditions such as pH, feeding materials, and nutritional compositions [5, 6], changes in reticular pH and bacterial communities following a high-grain diet are not fully understood. Therefore, the objective of this study was to identify the continuous changes in reticular pH and its effect on bacterial community structure following a high-grain diet simultaneously with those in the rumen.

## Results

### Ruminal and reticular pH and volatile fatty acids (VFAs)

**Fig. 1:**
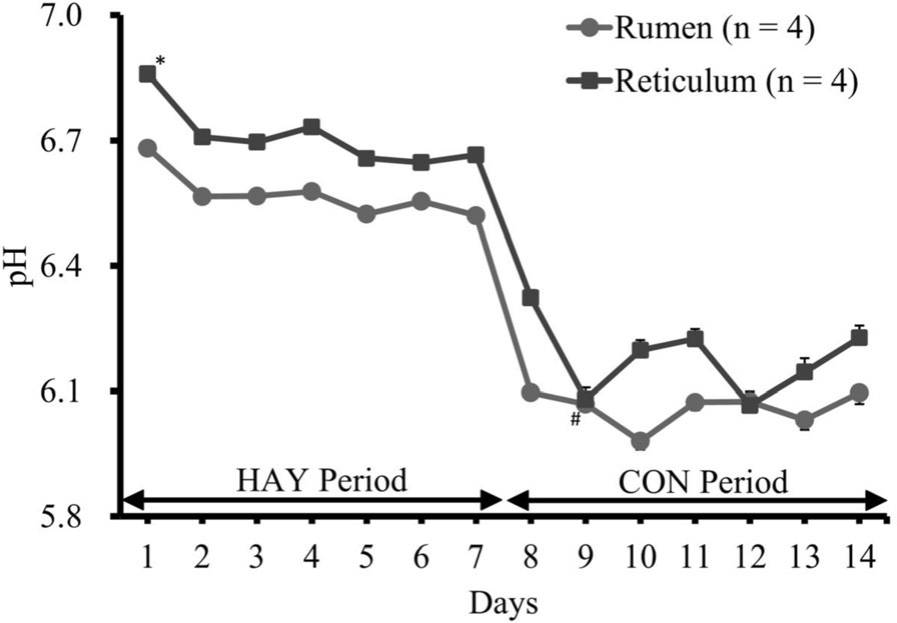
Daily changes in 24-h mean ruminal and reticular pH during the HAY and CON periods. Ruminal and reticular pH were measured simultaneously during the experimental period. Cattle were fed a high-forage diet for 7 days (HAY period) and then a high-grain diet for 7 days (CON period). A SARA challenge was performed as a high-forage period followed by a high-grain period. ^*^ denotes difference (*P* < 0.05) between the rumen and reticulum at the same day point. ^#^ denotes difference (*P* < 0.05) between the last day of the HAY period (day 7) and other day points at the same location. The values shown are means ± SEM

**Fig. 2:**
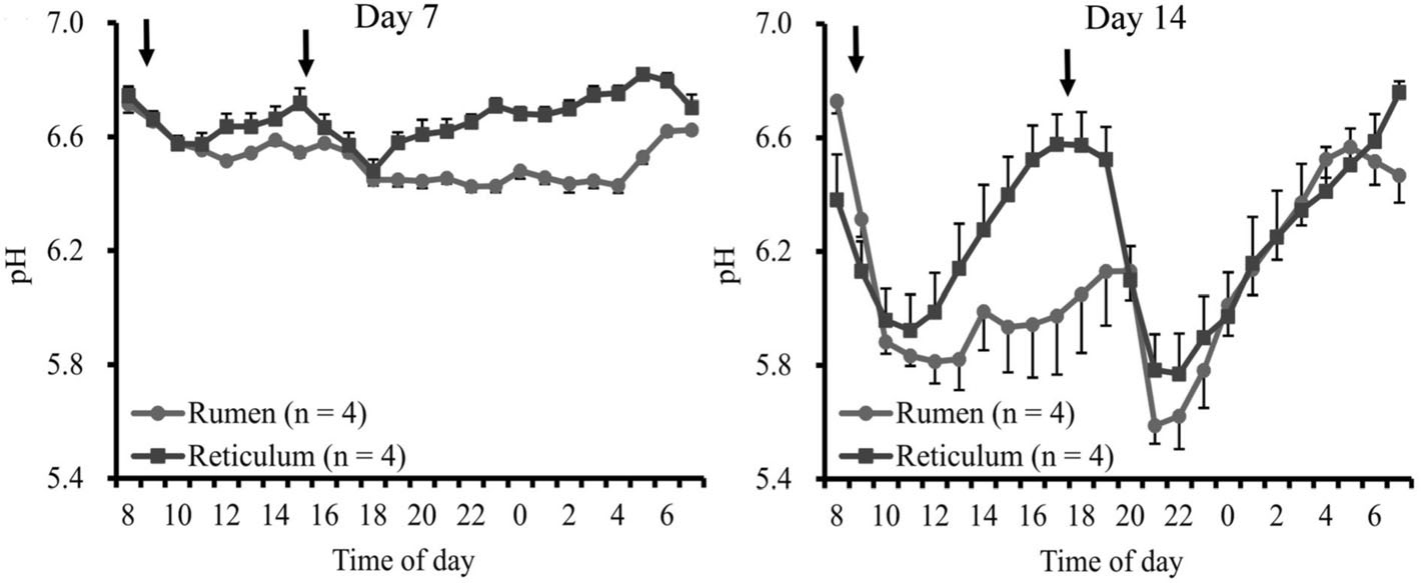
Diurnal changes in the 1-h mean ruminal and reticular pH. Ruminal and reticular pH were measured simultaneously during the experimental period. Cattle were fed a high-forage diet for 7 days (HAY period) and then a high-grain diet for 7 days (CON period). A SARA challenge was performed as a high-forage period followed by a high-grain period. Day 7 and 14 indicate the last days of the HAY (day 7) and CON (day 14) periods, respectively. Arrows indicate feeding times (08:00 and 17:00). The values shown are means ± SEM

**Fig. 3:**
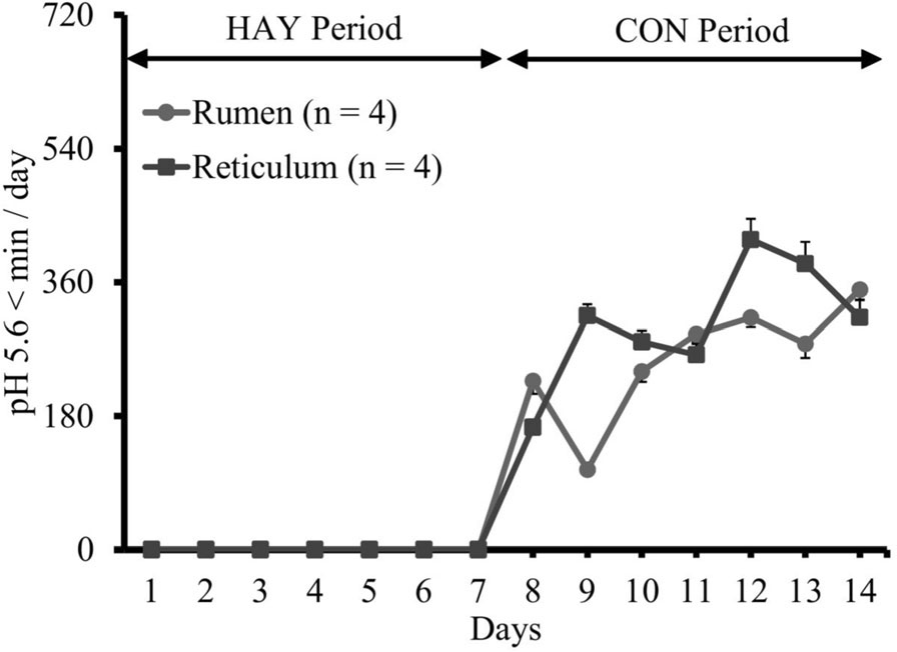
Daily changes in the 24-h mean time that ruminal and reticular pH < 5.6. Ruminal and reticular pH were measured simultaneously during the experimental period. Cattle were fed a high-forage diet for 7 days (HAY period) and then a high-grain diet for 7 days (CON period). A SARA challenge was performed as a high-forage period followed by a high-grain period. The values shown are means ± SEM

The 24-h mean ruminal and reticular pH decreased following the high-grain challenge (Fig. 1), and reticular pH was higher than ruminal pH during the HAY and CON periods, except on day 12. No significant differences were observed in 1-h mean pH and duration of pH < 5.6 in the rumen and reticulum. A significant difference (*P* < 0.05) between the ruminal and reticular pH was identified only on day 1, and ruminal pH was significantly (*P* < 0.05) decreased on day 9 compared to that on day 7 (Fig. 1). Linear regression analysis revealed a significant relationship between the 24-h mean pH and days in both rumen and reticulum (R^2^ = 0.79 and R^2^ = 0.81, respectively; *P* < 0.05). Diurnal changes in the 1-h mean ruminal and reticular pH observed on the last days of the HAY (day 7) and CON (day 14) periods are shown in Fig. 2. In day 14, linear regression analysis revealed that the ruminal and reticular pH decreased up to 4 h after the morning feeding (R^2^ = 0.92 and R^2^ = 0.89, respectively; *P* < 0.05 and *P* = 0.057, respectively), and increased from 4 h after the evening feeding (R^2^ = 0.99 and R^2^ = 0.97, respectively; *P* < 0.05). The duration of time where ruminal and reticular pH < 5.6 were 350 ± 14 and 312 ± 24 min/d, respectively, on the last day of the CON period (Fig. 3).

No significant differences were observed in total VFA and NH_3_-N concentrations in the rumen and reticulum (Table 1). However, a lower proportion of acetic acid and higher proportions of butyric acid and other VFAs (95% confidence interval: 2.14 to 3.46), excluding acetic acid, propionic acid, and butyric acid from the total VFAs, were observed during the CON period compared with the HAY period. The proportions of ruminal acetic acid and propionic acid were significantly lower and higher compared with the reticulum values, respectively, in both periods (*P* < 0.05). The proportions of ruminal butyric acid and other VFAs were significantly higher in the CON than in the HAY period (*P* < 0.05). The ratios of ruminal acetic acid to propionic acid in both periods were significantly lower than those in the reticulum (*P* < 0.05). The concentration of ruminal lactic acid was significantly higher in the CON than in the HAY period (*P* < 0.05).

**Table 1:** Total VFA, NH_3_-N, and lactic acid concentrations, individual VFA proportions, and acetic acid-to-propionic acid ratio

Items	HAY period		CON period		SEM
	Rumen	Reticulum	Rumen	Reticulum	
Total VFA, mmol/dL					
08:00	12.21	8.44	9.91	9.47	0.83
14:00	9.05	9.48	11.60	11.07	0.70
20:00	8.94	9.66	9.70	9.18	0.40
Acetic acid, %					
08:00	73.58^a^	75.39	61.12^A^	66.45	0.53
14:00	71.91^a^	73.98	57.23	60.87	1.22
20:00	70.52^A^	73.80	59.46^A^	63.11	1.36
Propionic acid, %					
08:00	17.68	16.73	17.49^A^	15.21	0.95
14:00	18.42	17.47	16.72	15.28	1.23
20:00	19.22^A^	17.35	18.33^A^	16.90	1.10
Butyric acid, %					
08:00	7.05	6.37^b^	17.07^A^	14.67	0.48
14:00	8.15^Aa^	7.27^b^	21.44	19.63	0.57
20:00	8.70	7.65	18.52^A^	16.73	0.59
Others, %					
08:00	1.70	1.51^B^	4.33	3.67	0.27
14:00	1.51^Aa^	1.28^B^	4.62^a^	4.21	0.30
20:00	1.57^A^	1.20^B^	3.68^a^	3.26	0.14
A/P Ratio					
08:00	4.16	4.52	3.58^A^	4.47	0.26
14:00	3.91	4.26	3.56	4.12	0.36
20:00	3.69^A^	4.27	3.31^A^	3.82	0.30
NH_3_-N, mg/dL					
08:00	9.02	8.17	5.62	6.44	0.61
14:00	4.75	5.21	3.46	2.95	0.51
20:00	4.70	5.18	5.98	6.33	0.79
Lactic acid, g/L					
08:00	0.018	0.028	0.014	0.015	0.006
14:00	0.017	0.016	0.015	0.012	0.004
20:00	0.010^A^	0.015	0.375	0.381	0.059

^A,B^ denotes significant difference (*P* < 0.05) between the rumen and reticulum at the same period

^a,b^ denotes significant difference (*P* < 0.05) between the HAY and CON periods at the same location

### Bacterial abundance

**Fig. 4:**
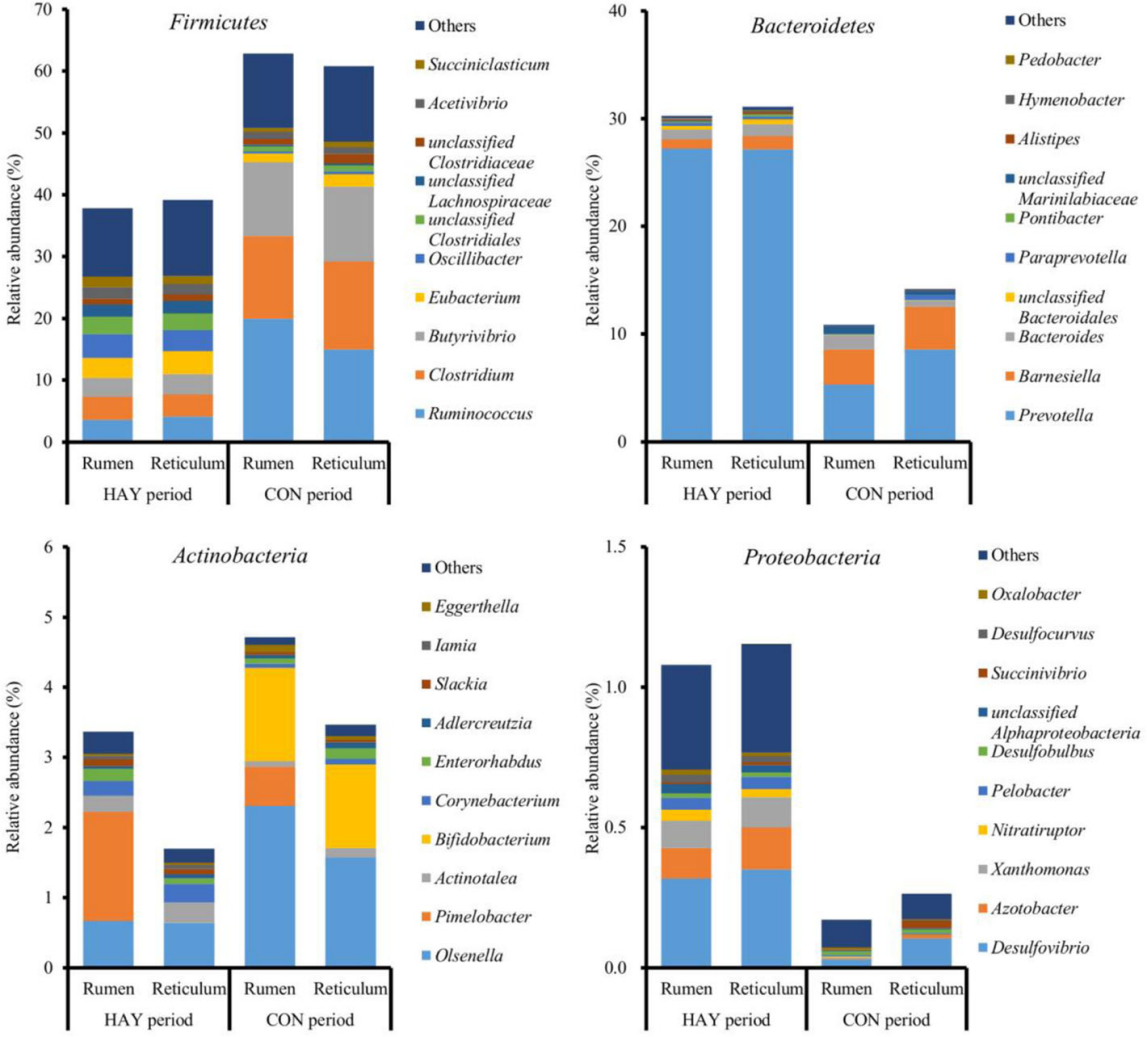
Relative abundances of the four major bacterial phyla and genus profiles. Pyrosequencing analysis was performed using rumen and reticulum fluid samples collected at 0800 h on the last days of the HAY and CON periods. Data are shown as the percentages of the total identified sequences per group

Of the major phyla, *Firmicutes*, *Bacteroidetes*, and *Actinobacteria* were the most abundant, accounting for 74.3% of the total ruminal sequences and 74.0% of the total reticular sequences (Fig. 4). The remaining phyla had low relative abundances of < 1%. A total of 337 bacterial genera were identified; the relative abundances of 323 genera comprised < 1% of the total sequences. Of the major genera, *Prevotella* was the most abundant in both the rumen and reticulum, followed by *Ruminococcus* and *Clostridium*. The relative abundance of *Prevotella* (18.4%) in the rumen was lower than that in the reticulum (21.2%), whereas the relative abundances of *Ruminococcus* (10.5%) and *Clostridium* (7.7%) in the rumen were higher than those of the reticulum (7.7% and 6.8%, respectively) regardless of diet.

At the phylum level, the relative abundances of *Firmicutes* and *Bacteroidetes* differed significantly between the HAY and CON periods (*P* < 0.05; Table 2). At the genus level, the relative abundances of *Prevotella*, *Eubacterium* (95% confidence interval: 2.02 to 3.18), *Oscillibacter*, and *Succiniclasticum* were lower during the CON period, whereas the relative abundances of *Ruminococcus*, *Clostridium*, and *Olsenella* (95% confidence interval: 0.78 to 1.80) were higher during this period; these genera differed significantly between the HAY and CON periods (*P* < 0.05; Table 2). The relative abundance of *Barnesiella* exhibited a trend for difference (*P* = 0.077) between the two periods, being higher during the CON period compared with the HAY period. Considering the relative abundance of *Eubacterium* spp., a lower relative abundance (*P* < 0.05) was detected for rumen samples than reticulum samples. The period × location interaction was significant (*P* < 0.05) for the relative abundance of *Olsenella*. The relative abundances of *Olsenella* in the rumen and reticulum during the CON period were significantly (*P* < 0.05) lower compared with those during the HAY period. During the CON period, the relative abundance of *Olsenella* was significantly (*P* < 0.05) higher in the rumen compared with the reticulum. However, there was no statistical significance in the relative abundances of *Butyrivibrio* (95% confidence interval: 3.20 to 12.0), unclassified *Clostridiales* (95% confidence interval: 0.73 to 2.93), *Acetivibrio* (95% confidence interval: 0.63 to 2.27), unclassified *Lachnospiraceae* (95% confidence interval: 0.33 to 1.95), unclassified *Clostridiaceae* (95% confidence interval: 0.81 to 1.45), and *Bacteroides* (95% confidence interval: 0.43 to 1.55) between the two periods and two locations.

**Table 2:** Relative abundance (% of total sequences) of the major bacterial phyla and genera

Items	HAY Period		CON Period		SEM	*P* value		
	Rumen (*n* = 4)	Reticulum (*n* = 4)	Rumen (*n* = 4)	Reticulum (*n* = 4)		Period (P)	Location (L)	P x L
Phylum								
*Firmicutes*	37.80	39.18	62.85	60.79	5.54	0.002	0.962	0.706
*Bacteroidetes*	30.26	31.11	10.84	14.19	5.89	0.014	0.762	0.822
*Actinobacteria*	3.36	1.70	4.72	3.46	1.16	0.114	0.401	0.814
Genus								
*Prevotella*	27.20	27.15	5.29	8.56	4.84	0.006	0.766	0.746
*Ruminococcus*	3.56	4.09	19.96	14.97	3.28	0.019	0.626	0.541
*Clostridium*	3.76	3.59	13.37	14.22	2.16	0.006	0.910	0.839
*Butyrivibrio*	3.05	3.28	11.95	12.13	3.19	0.117	0.931	0.977
*Eubacterium*	3.24	3.75	1.41	1.98	0.34	0.025	0.027	0.912
*Oscillibacter*	3.84	3.40	0.34	0.49	0.46	0.001	0.825	0.608
unclassified *Clostridiales*	2.85	2.71	0.81	0.97	0.94	0.258	0.426	0.692
*Barnesiella*	0.88	1.22	3.25	3.97	1.11	0.077	0.743	0.879
*Acetivibrio*	1.89	1.66	1.11	1.12	0.87	0.716	0.459	0.996
unclassified *Lachnospiraceae*	1.95	2.01	0.28	0.30	0.59	0.173	0.593	0.431
*Succiniclasticum*	1.70	1.31	0.65	0.90	0.26	0.037	0.800	0.280
*Olsenella*	0.67^a^	0.63^b^	2.31^ac^	1.57^bc^	0.39	0.012	0.144	0.024
unclassified *Clostridiaceae*	0.95	1.09	0.96	1.53	0.31	0.800	0.265	0.552
*Bacteroides*	0.91	1.12	1.36	0.57	0.49	0.519	0.985	0.782

^a-a, b-b, c-c^ means within a row, same superscripts differ significantly (*P* < 0.05)

### Bacterial diversity analysis

**Fig. 5:**
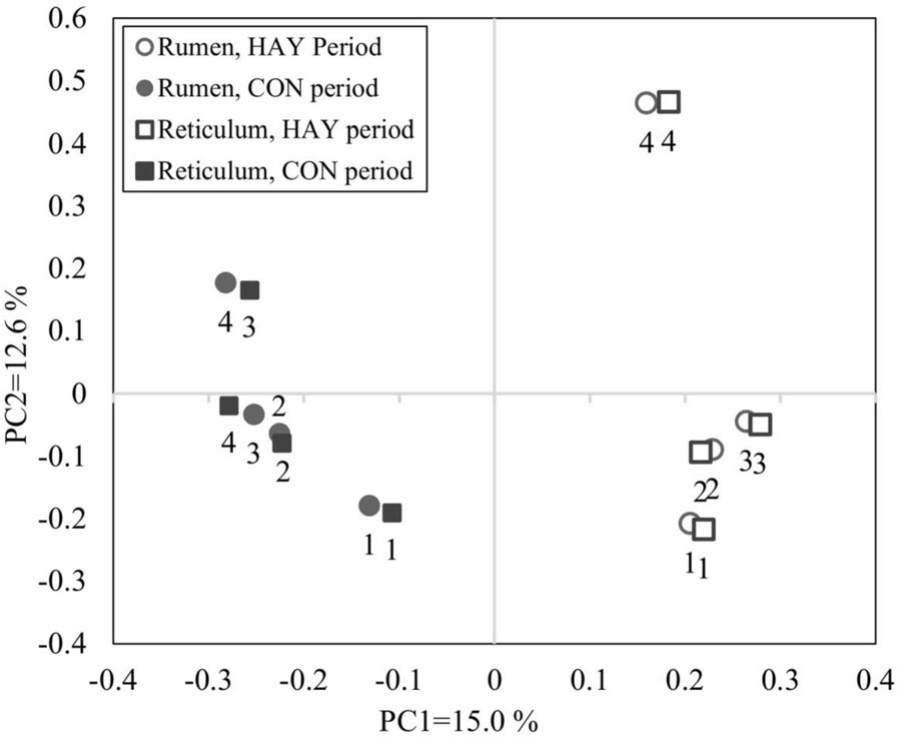
Principal coordinates analysis (PCoA) plots generated from the 454 pyrosequencing data. Pyrosequencing analysis was performed using rumen and reticulum fluid samples collected at 0800 h on the last days of the HAY and CON periods. PC1 and PC2 represent principal components 1 and 2, respectively. The numbers represent the individual cattle

Rarefaction curves calculated at a 97% similarity level indicated that the reticulum had higher bacterial diversity than did the rumen (Additional file 1: Figure S1), and the HAY period had higher bacterial diversity than did the CON period, regardless of the location (Table 3). The PCoA results indicated that the HAY period was separate from the CON period in the plot regardless of location, whereas the individual plots for cattle showed close similarity between the rumen and reticulum (PC1 + PC2 = 27.6%; Fig. 5). Moreover, the operational taxonomic units (OTUs), abundance-based coverage estimator (ACE), Chao1, Shannon (95% confidence interval: 4.70 to 5.52), Simpson indices were higher in the HAY period, while Simpson index was higher in the CON period. The effect of location and the interaction effect of period × location were not significant for indices.

**Table 3:** Bacterial diversity calculated from 454 pyrosequencing data

Items	HAY Period		CON Period		SEM	*P* value		
	Rumen (*n* = 4)	Reticulum (*n* = 4)	Rumen (*n* = 4)	Reticulum (*n* = 4)		Period (P)	Location (L)	P x L
OTUs	993	1115	582	632	106	< 0.001	0.587	0.604
ACE	2542	3057	1347	1669	234	< 0.001	0.242	0.563
Chao1	1774	2195	1003	1202	186	< 0.001	0.279	0.430
Shannon	5.60	5.85	4.49	4.50	0.28	< 0.001	0.613	0.455
Simpson	0.016	0.012	0.057	0.062	0.01	0.010	0.963	0.717

### Copy numbers of bacterial 16S genes

The copy numbers of total methanogens, *Fibrobacter succinogenes*, and *Selenomonas ruminantium* differed significantly (*P* < 0.05) between the HAY and CON periods, being higher during the HAY period (Table 4). The copy number of *Ruminococcus albus* was tended (*P* = 0.068) to be higher during the HAY period compared with the CON period. No statistical difference was identified in the copy numbers of *Megasphaera elsdenii* (95% confidence interval: 2.93 to 4.87), *Streptococcus bovis* (95% confidence interval: 5.65 to 7.47), and *Ruminococcus flavefaciens* (95% confidence interval: 5.92 to 7.042). The copy number of total methanogens was significantly different between the rumen and reticulum (*P* < 0.05), being higher in the rumen. The period × location interaction was significant for *Ruminococcus flavefaciens* (*P* < 0.05). The copy number of *Ruminococcus flavefaciens* in the rumen during the HAY period was significantly (*P* < 0.05) higher compared with that during the CON period.

**Table 4:** Copy number of *mcr*A and 16S rRNA genes identified form qRT-PCR

Items	HAY Period		CON Period		SEM	*P* value		
	Rumen (*n* = 4)	Reticulum (*n* = 4)	Rumen (*n* = 4)	Reticulum (*n* = 4)		Period (P)	Location (L)	P x L
Total methanogens	5.78	5.34	4.73	3.97	0.23	0.004	0.018	0.583
*Fibrobacter succinogenes*	5.88	4.41	3.45	2.25	0.48	< 0.001	0.119	0.600
*Megasphaera elsdenii*	2.97	2.89	3.76	5.99	0.64	0.180	0.741	0.230
*Streptococcus bovis*	7.00	6.74	4.89	7.62	0.55	0.465	0.279	0.279
*Ruminococcus albus*	6.13	4.65	4.76	4.45	0.37	0.068	0.123	0.147
*Ruminococcus flavefaciens*	7.60^a^	6.03^b^	5.87^a^	6.42^c^	0.45	0.270	0.582	0.019
*Selenomonas ruminantium*	7.20	6.65	6.21	6.11	0.24	0.041	0.135	0.481

^a-a^ means within a row, same superscripts differ significantly (*P* < 0.05)

## Discussion

This study aimed to identify the continuous changes in reticular pH following a high-grain diet and to investigate its effect on the reticular bacterial composition, diversity, and similarity simultaneously with those in the rumen. Sato et al. [2] previously identified significant positive correlations between reticular and ruminal pH in both healthy and SARA-induced cows. SARA was diagnosed based on a condition characterized by ruminal pH < 5.6 for more than 3 h per day [1] in our study, and the ruminal and reticular pH remained under 5.6 over 3 h from 2 days after the high-grain diet feeding to the end of the experiment. Therefore, SARA was successfully induced in the present study. Furthermore, the lower proportion of acetic acid and higher proportion of butyric acid following the high-grain diet were consistent with a previous study [7]. Although a temporarily higher concentration of lactic acid at 20:00 was identified in the CON period, this increase may not accurately reflect the diurnal changes in the 1-h mean ruminal and reticular pH throughout a day. Therefore, VFA production by microbes and its removal by absorption through ruminal epithelial cells, neutralization with salivary buffer, and passage to the lower digestive tracts [10] might be a more plausible determinant of the ruminal and reticular pH. However, no statistically significant differences in total VFA concentration were identified, so we can only speculate that fatty acid removal was encouraged by the marked decrease in ruminal and reticular pH in this study.

In our study, significant change in the relative abundance at the phylum level was only identified between the HAY and CON periods. *Firmicutes* and *Bacteroidetes* were the most abundant bacterial phyla in both the rumen and reticulum, and they were affected by a high-grain diet in our study. Gram-negative *Bacteroidetes* bacteria have been shown to decrease in the rumen of cattle with SARA induced by either grain or alfalfa pellets [11], and their relative abundance was reduced in cattle with induced SARA [5]. Because a low rumen pH can lead to death and lysis of gram-negative bacteria [9], higher acidity during the CON period may cause a decrease in the relative abundance of *Bacteroidetes*. The gram-positive bacteria phylum *Firmicutes* is composed of species metabolically capable of consuming newly available fermentable carbohydrates [9]; therefore, an increase in their relative abundance may be related to the starch-rich high-grain diet and the decrease in the relative abundance of *Bacteroidetes* during the CON period.

At the genus level, changes in relative abundance were primarily identified between the high-forage and high-grain diets. *Prevotella* bacteria can use a variety of substrates in the rumen [12, 13], and was the predominant genus in our study. The lower relative abundance of *Prevotella* observed during the CON period is consistent with previous studies demonstrating that starch addition lowers the relative abundance of *Prevotella* [4]. Although the relationship between substrate and growth of *Prevotella* is not well studied, a higher acidity during the CON period may affect the relative abundance of *Prevotella*. By contrast, the relative abundance of *Ruminococcus* was higher during the CON period. Although *Ruminococcus* is commonly known for cellulolytic species, several others (e.g., *Ruminococcus bromii*) can exploit starch [14]. Therefore, the increase in the relative abundance of *Ruminococcus* during the CON period may be related to an increase in starch-fermenting *Ruminococcus* species. In addition, the genus *Clostridium* involved in the digestion of various substrates in the rumen [15] was higher during the CON period than during the HAY period, and it was consistent with a previous study [5].

Previously, Taguchi et al. [16] reported that *Eubacterium ruminantium* is associated with hemicellulose (mainly xylan) degradation in the rumen, and lower relative amount of *Eubacterium ruminantium* in the concentrate diet than the hay diet Tajima et al. [17] was consistent with the present study. In addition, *Eubacterium* was the only genus that differed significantly between the rumen and reticulum in this study. However, the only difference between the rumen and reticulum conditions that we suggest in this study was a slightly higher reticular pH than ruminal pH throughout the experiment. Although, the xylanase activity of *Eubacterium ruminantium* decreased in accordance with a decrease in pH (from 7.0 to 5.0; [16]), this could not be linked directly to the relative abundance of *Eubacterium* genus. Therefore, further studies are required to clarify the effects of acidic conditions on the changes in the relative abundance of *Eubacterium* genus. Collectively, these results suggest that changes in the relative abundances of bacterial genera may be mainly influenced by changes in useable substrates, as well as by the acidic environment.

The passage rate of ruminal contents from the rumen to the duodenum is increased in cattle by feeding a concentrated diet, resulting in less time for microbial fermentation [18]. The increased passage rate could cause methanogenesis to shift to the hind gut and manure [19], and it was consistent with the lower copy number of total methanogens in the CON period. In this study, several bacteria well known for their cellulolytic activity (*Fibrobacter succinogenes*, *Ruminococcus albus*, and *Ruminococcus flavefaciens*; [20]) had higher copy numbers during the HAY period, which may be related to the higher fiber contents during this period. Meanwhile, *Megasphaera elsdenii*, a lactate-metabolizing species, increases in abundance as the bacterial community adapts to more readily fermentable carbohydrates [21], and the higher copy number of *Megasphaera elsdenii* during the CON period may be associated with higher relative abundance of lactate-producing bacterium, such as *Olsenella*, during the same period. Moreover, dilution of the reticulum contents occurs with fresh and less-fermented feed [2, 3], which might cause the lower bacterial copy numbers in the reticulum compared with the rumen.

Feeding a high-grain diet to cattle decreases the ruminal pH and bacterial diversity of the rumen epithelial community [7], and calves fed a high-grain diet show significantly lower ruminal pH and bacterial diversity compared with calves fed a high-grain diet with forage [6]. In the present study, ruminal and reticular pH decreased after cattle transitioned from a high-forage to a high-grain diet, and bacterial diversity indices, such as OTUs, ACE, Chao1, and Shannon index, were lower during the CON period than during the HAY period. Furthermore, PCoA results showed that the HAY period was separate from the CON period in the plot regardless of location, and the rarefaction curve was consistent with the bacterial diversity analysis. Therefore, we assumed that the lower ruminal and reticular pH during the CON period resulted in the lower bacterial diversity. Although no statistically significant difference was identified in bacterial diversity between the rumen and reticulum, the higher diversity indices identified in the reticulum were consistent with the higher pH there compared to that in the rumen, which supports our hypothesis.

## Conclusion

To the best of our knowledge, this is the first study to examine changes in reticular pH continuously and bacterial community structure following a high-grain diet feeding. Ruminal and reticular pH decreased after feeding a high-grain diet, and SARA was successfully induced during the CON period. Changes in the bacterial community structure and copy number were mainly identified between the two periods, which might be related to the changes in pH and diet. Furthermore, the decrease in pH identified during the CON period was consistent with the low bacterial diversity during the same period. These findings suggest that bacterial composition and diversity in both the rumen and reticulum were affected mainly by the acidic conditions and substrates useable for their growth, all of which may influence the fermentative ability of the rumen and reticulum.

## Methods

### Animals and experimental design

All animals were cared for according to protocols approved by Iwate University Laboratory Animal Care and Use Committee (A201401). Four rumen-cannulated Holstein bull cattle (192 ± 12 kg; 9.0 ± 1.4 months of age) were used in this study. Following the 7-day high-forage adaptation period, all cattle were fed a high-forage diet for 7 days (HAY period), and then a high-grain diet for 7 days (CON period). A SARA challenge was defined a HAY period followed by a CON period. During the experimental periods, cattle were fed mixed hay (orchard and timothy hay) in the HAY period and a high-grain diet with a forage-to-concentrate ratio of 19:81 (dry matter basis) during the CON period. The diet was supplied daily in two equal portions at 08:00 and 17:00. All cattle had free access to water and mineral salt block (E100TZ; ZENOAQ, Koriyama, Fukushima, Japan) throughout the study period. Daily total DMI was recorded for individual cattle throughout the experimental period, and all feed offered to the cattle was consumed. The amount and content of a high-forage (control) and a high-grain (SARA-inducing) diet were based on our previous study [8]. The chemical compositions of the mixed hay and high-grain diet fed to the cattle are shown in Additional file 1: Table S1.

### Sampling, measurements, and diagnosis of SARA

Ruminal and reticular pH were simultaneously measured every 10 min using a radio transmission system (YCOW-S; DKK-TOA Yamagata, Yamagata, Japan), as reported previously [22]. The pH sensors were placed in the ventral sac of the rumen and the reticulum through the rumen fistula. Ruminal and reticular pH were continuously measured for 7 days during the HAY period (days 1–7) and consecutively for 7 days during the CON period (days 8–14). Using the rumen and reticulum accessible tubes, the fluid samples were collected from the rumen and reticulum adjacent to the pH sensor at 8:00, 14:00, and 20:00 on the last days of the HAY (day 7) and CON (day 14) periods, respectively.

The fluid samples were filtered immediately through two layers of cheesecloth after sampling. For the VFA analysis, 1 mL 25% HO_3_P in 3 N H_2_SO_4_ was added to 5 mL rumen and reticulum fluid. Total VFA and VFA components (i.e., acetic acid, propionic acid, and butyric acid) were separated and quantified by gas chromatography (Model 135, Hitachi, Tokyo, Japan) using a packed glass column (Thermon-3000, 3%) on a Shimalite TPA 60–80-mesh support (Shinwa Chemical Industries, Ltd., Kyoto, Japan). For the lactic acid analysis, the fluid samples were centrifuged at 2000×g for 15 min, and concentration in the supernatant were determined using a commercially available kit (F-kit; D-lactate/L-lactate, J. K. International Co., Tokyo, Japan). To measure NH_3_-N concentration, fluid samples were analyzed using the steam distillation method with an NH_3_-N analyzer (Kjeltec Auto Sampler System 1035 Analyzer, Tecator, Sweden).

### DNA isolation

Total bacterial DNA was extracted as described previously [6]. Briefly, the fluid samples were incubated with 750 μg/mL lysozyme (Sigma-Aldrich, St. Louis, MO, USA) at 37 °C for 90 min. Then, 10 μL purified achromopeptidase (Wako Pure Chemical Industries, Ltd., Osaka, Japan) was added at a concentration of 10,000 U/mL and incubated at 37 °C for 30 min. The suspension was treated with 60 μL 1% sodium dodecyl sulfate and 1 mg/mL proteinase K (Merck Japan, Tokyo, Japan), and incubated at 55 °C for 5 min. The lysate was treated with phenol/chloroform/isoamyl alcohol (Wako Pure Chemical Industries, Ltd.) and chloroform (Life Technologies Japan, Ltd., Tokyo, Japan). DNA was precipitated by adding 5 M NaCl and 100% ethanol and centrifuged at 21,900×*g* for 15 min. The DNA pellet was rinsed with 70% ethanol, dried, and dissolved in TE buffer. The purified DNA was quantified using a Biospec-nano (Shimadzu, Kyoto, Japan) and stored at − 80 °C until further analysis.

### DNA pyrosequencing

The V1/V2 region of the 16S rRNA gene was amplified using a forward primer (5′-CCATCTCATCCCTGCGTGTCTCCGACTCAGNNNNNNNNNNAGRGTTTGATYMTGGCTCAG-3′) containing 454 primer A, a unique 10-bp barcode sequence for each sample (indicated as N), and 27Fmod (5′-AGRGTTTGATYMTGGCTCAG) in which the third base, A, in the original primer 27F was changed to R, as well as the reverse primer (5′-CCTATCCCCTGTGTGCCTTGGCAGTCTCAGTGCTGCCTCCCGTAGGAGT-3′) containing 454 primer B and reverse primer 338R (5′-TGCTGCCTCCCGTAGGAGT). Amplified products of ~ 370 bp were confirmed using agarose gel electrophoresis, purified using AMPure XP magnetic purification beads (Beckman Coulter, Inc., Brea, CA, USA), and quantified using the Quant-iT PicoGreen dsDNA Assay Kit (Life Technologies Japan). Mixed samples were prepared by pooling approximately equal amounts of PCR amplicons from each sample, and then subjected to 454 GS Junior (Roche Applied Science, Indianapolis, IN, USA) sequencing following the manufacturer’s instructions.

### Pyrosequencing data analysis

All pyrosequencing reads were filtered according to the procedure of Kim et al. [23], who developed an analysis pipeline for barcoded 454 pyrosequencing of PCR amplicons in V1/V2, the region amplified by the 27Fmod/338R primers. A total of 151,797 filter-passed reads were processed using MOTHUR (ver. 1.35, University of Michigan; http://www.mothur.org/wiki/; [24]), and all samples were standardized by random subsampling to 4522 sequences per sample using the “sub.sample” command to generate rarefaction curves and calculate ACE, Chao1 richness estimator, and Shannon diversity index, according to the Illumina MiSeq protocol described previously [25]. Unique sequences were determined and used to align against the SILVA reference alignment database [26]; chimera were removed using chimera.uchime (http://drive5.com/uchime); sequences identified as being of eukaryotic origin were removed; the candidate sequences were screened and preclustered to eliminate outliers; and a distance matrix was generated from the resulting sequences. Sequences were clustered into OTU, with a cutoff of 97% similarity. A rarefaction curve was generated at the 97% similarity level, which was calculated by the distance-based OTU [27]. To calculate the nonparametric species richness estimators Chao 1 and ACE and the Shannon diversity index, the “summary.single” command was used. The unweighted UniFrac distance method [28] was used to perform a principal coordinates analysis (PCoA) with all OTU.

### Real-time quantitative PCR

Quantitative real-time PCR (qRT-PCR) was performed to evaluate the copy number of methyl-coenzyme M reductase α-subunit (*mcrA*) from total methanogens, and 16S rRNA genes from *Fibrobacter succinogenes*, *Megasphaera elsdenii*, *Ruminococcus albus*, *Ruminococcus flavefaciens*, *Streptococcus bovis*, and *Selenomonas ruminantium* using SYBR green (iQ SYBR Green Supermix, Bio-Rad, Hercules, CA, USA) with the MiniOpticon Real-Time PCR system (Bio-Rad). Primer pairs (Additional file 1: Table S2) were selected to detect bacterial species closely associated with dietary changes and other bacterial species. Each sample contained 10 ng DNA, 2× SYBR green, and 0.6 μM each primer in a final volume of 20 μL. Amplification conditions were as follows: 95 °C for 3 min; 40 cycles of 10 s at 95 °C; 20 s at 63 °C (for total methanogens), 60 °C (for *Fibrobacter succinogenes*), 58 °C (for *Megasphaera elsdenii*), 57 °C (for *Streptococcus bovis* and *Selenomonas ruminantium*), or 55 °C (for *Ruminococcus albus* and *Ruminococcus flavefaciens*); and 30 s at 72 °C. The fluorescence signal was collected at the end of each cycle. To obtain melting curve data, the temperature was increased in 0.5 °C increments from 65 to 94 °C. A standard curve for each primer pair was constructed from recombinant plasmid DNA containing 16S rRNA inserts of DNA purified from a pure culture of the target species. The strains used for plasmid preparation were as follows: *Methanobrevibacter ruminantium* JCM13430 (DSM1093), *Fibrobacter succinogenes* ATCC19169, *Megasphaera elsdenii* ATCC25940, *Ruminococcus albus* ATCC27210, *Ruminococcus flavefaciens* ATCC19208, *Streptococcus bovis* ATCC33317, and *Selenomonas ruminantium* ATCC12561. Plasmid DNA was quantified and subjected to seven sequential 10-fold dilutions. Data were collected and processed using CFX Manager software ver. 1.5 (Bio-Rad).

### Statistical analyses

Ruminal and reticular pH data and the duration of pH < 5.6 were summarized as 24-h means during the HAY and CON periods. The normality of the distribution of variables was tested using Shapiro-Wilk test, and non-normal data (the proportion of other VFAs, Shannon index, the relative abundances of *Butyrivibrio*, *Eubacterium*, unclassified *Clostridiales*, *Acetivibrio*, unclassified *Lachnospiraceae*, *Olsenella*, unclassified *Clostridiaceae*, *Bacteroides*, and the copy numbers of *Megasphaera elsdenii*, *Streptococcus bovis*, and *Ruminococcus flavefacience*) were root-square transformed before analysis. One-way repeated measures ANOVA followed by Tukey multiple comparison method was used to determine the significance of the difference in 24-h and 1-h means pH, the duration of pH < 5.6, total VFA concentration, the proportions of individual VFA, and the concentrations of NH_3_-N and lactic acid to compare rumen × reticulum in a same period and HAY × CON periods in a same location. The relative abundances of bacteria phyla and genera, bacterial diversity indices, and bacterial species copy numbers were analyzed using two-way repeated-measures ANOVA and Tukey’s multiple range tests. The statistical model included the fixed effects of location (rumen and reticulum), period (HAY and CON periods), and their interactions, plus the random effect of animal. Linear regression analysis of 24-h and 1-h mean ruminal and reticular pH against days and hours was performed to determine whether there are significant changes in pH during the experimental period. All numerical data were analyzed using Prism ver. 7.01 (GraphPad Software, Inc., La Jolla, CA, USA), and are expressed as means ± standard error of mean (SEM). Differences were considered significant at *P* < 0.05, and trends suggesting possible significance were determined at 0.05 ≤ *P* < 0.10.

## Additional file

Additional file 1: **Table S1.** Compositions of the high-forage and high-grain diet on percentage (%) and dry matter bases. **Table S2.** Primers sequences used for qRT-PCR. **Figure S1.** Rarefaction curves calculated from the 454 pyrosequencing data at a 97% similarity level in the rumen and reticulum. Cattle were fed a high-forage diet until day 7 (HAY period) and a high-grin diet until day 14 (CON period). Solid and dash dot lines represent the rumen of the HAY and CON periods, respectively. Dash and dot lines represent the reticulum of the HAY and CON periods, respectively. (DOCX 441 kb)

## Abbreviations

ACE: Abundance-based coverage estimator
ANOVA: Analysis of variance
OTU: Operational taxonomic unit
PC: Principal components
PCoA: Principal coordinate analysis
qRT-PCR: Quantitative real time polymerase chain reaction
SARA: Subacute ruminal acidosis
SE: Standard error
VFA: Volatile fatty acid
