# Supplementary material for: Changes in ruminal and reticular pH and bacterial communities in Holstein cattle fed a high-grain diet
Source: BMC Vet Res. 2018 Oct 12;14:310. doi: 10.1186/s12917-018-1637-3 (PMC6186129; doi:10.1186/s12917-018-1637-3)
Supplement: Supplementary file 1 — Table S1. Compositions of the high-forage and high-grain diet on percentage (%) and dry matter bases. Table S2. Primers sequences used for qRT-PCR. Figure S1. Rarefaction curves calculated from the 454 pyrosequencing data at a 97% similarity level in the rumen and reticulum. Cattle were fed a high-forage diet until day 7 (HAY period) and a high-grin diet until day 14 (CON period). Solid and dash dot lines represent the rumen of the HAY and CON periods, respectively. Dash and dot lines represent the reticulum of the HAY and CON periods, respectively. (DOCX 441 kb) [file 12917_2018_1637_MOESM1_ESM.docx]

**Additional file 1: SUPPLEMENTARY TABLE AND FIGURE LEGENDS**

**Table S1.** Compositions of the high-forage and high-grain diet on percentage (%) and dry matter bases

| Composition | High-forage diet  (HAY period) | High-grain diet  (CON period) |
| --- | --- | --- |
|  |  |  |
| Amount (%) |  |  |
| Orchard and timothy hay | 100 | 18.75 |
| Concentrate | 0 | 60.94 |
| Corn flake | 0 | 20.31 |
| Dry matter (DM) | 87.2 | 89.1 |
| DM basis (%) |  |  |
| TDN^1^ | 60.9 | 80.1 |
| Crude protein | 13 | 17 |
| Crude fat | 1.3 | 3.6 |
| ADF^2^ | 40.5 | 13.8 |
| NDF^3^ | 68 | 26.7 |
| Ca^4^ | 0.5 | 0.8 |
| P^5^ | 0.3 | 0.5 |
| ^1^Total Digestible Nutrients, ^2^Acid Detergent Fiber, ^3^Neutral Detergent Fiber, ^4^Calcium, ^5^Phosphate | | |

**Table S2.** Primers sequences used for qRT-PCR

| Targets | Primer sequences (5'-3') | | Annealing  Temperature (℃) | Reference |
| --- | --- | --- | --- | --- |
|  |  |  |  |  |
| Total methanogens | F | GGTGGTGTMGGDTTCACMCARTA | 63 | Steinberg and Regan, 2008 |
|  | R | CGTTCATBGCGTAGTTVGGRTAGT |  |  |
| *Fibrobacter succinogenes* | F | GGTATGGGATGAGCTTGC | 60 | Tajima et al., 2001 |
|  | R | GCCTGCCCCTGAACTATC |  |  |
| *Megasphaera elsdenii* | F | TGCTAATACCGAATGTTG | 57 | Tajima et al., 2001 |
|  | R | TCCTGCACTCAAGAAAGA |  |  |
| *Ruminococcus albus* | F | CCCTAAAAAGCAGTCTTAGTTCG | 55 | Koike and Kobayashi, 2001 |
|  | R | CCTCCTTGCGGTTAGAACA |  |  |
| *Ruminococcus flavefaciens* | F | TCTGGAAACGGATGGTA | 55 | Koike and Kobayashi, 2001 |
|  | R | CCTTTAAGACAGGAGTTTACAA |  |  |
| *Streptococcus bovis* | F | CTAATACCGCATAACAGCAT | 57 | Tajima et al., 2001 |
|  | R | AGAAACTTCCTATCTCTAGG |  |  |
| *Selenomonas ruminantium* | F | GACCGAAACTGCGATGCTAGA | 58 | Ouwerker et al., 2002 |
|  | R | CGCCTCAGCGTCAGTTGTC |  |  |

F, forward; R, reverse

**Figure S1.**


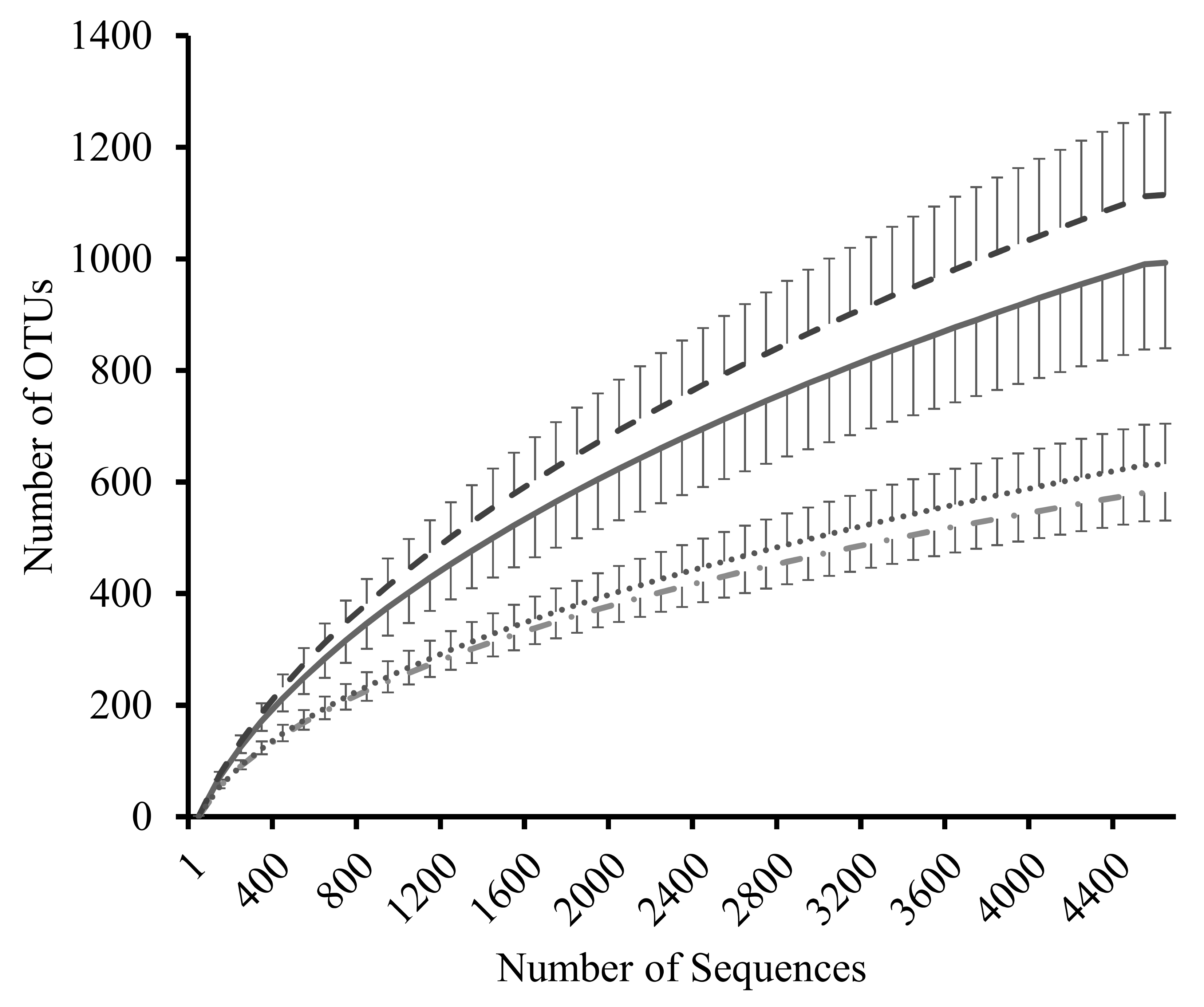


**Figure S1.** Rarefaction curves calculated from the 454 pyrosequencing data at a 97% similarity level in the rumen and reticulum. Cattle were fed a high-forage diet until day 7 (HAY period) and a high-grin diet until day 14 (CON period). Solid and dash dot lines represent the rumen of the HAY and CON periods, respectively. Dash and dot lines represent the reticulum of the HAY and CON periods, respectively.
